# Altered behavior with an organic cause: a case report

**DOI:** 10.1186/s13256-019-2112-x

**Published:** 2019-06-25

**Authors:** Dolores Mejía, Rafael Contreras, Guarina Molina, Michael Alcántara

**Affiliations:** 1 Research Department, General Hospital of the Health Plaza (HGPS), Santo Domingo, Dominican Republic; 2 Universidad Iberoamericana, Santo Domingo, Dominican Republic

**Keywords:** Insulinoma, Neuroendocrine tumor, Altered mental status

## Abstract

**Background:**

Insulinomas are pancreatic endocrine tumors of rare incidence worldwide, the vast majority are of single occurrence and benign. These may not always present with the clear symptoms described in the literature and may be overlooked because their neuroglycopenic characteristics present in a fashion similar to some psychiatric conditions.

**Case report:**

A 50-year-old Hispanic man referred severe psychomotor symptoms, described as anxiety, aggressiveness, agitation, weakness, diaphoresis, and decreased visual acuity. Laboratory testing performed during his last episode revealed increased insulin levels and C-peptide among other findings. Imaging, biopsy, and histopathologic analysis confirmed an insulinoma was the cause of the symptoms, proving the importance of ruling out organic causes of altered mental status prior to consideration of psychiatric disorders.

**Conclusion:**

It is of critical importance to rule out organic causes of altered mental status prior to consideration of psychiatric disorders, as unusual diseases may be overlooked by physicians and be detrimental to the patient’s progress.

## Background

The diagnosis of diseases of low incidence is the most challenging for physicians, especially when their clinical manifestations are very similar to other pathologies with higher incidence and prevalence. Insulinomas represent over 50% of pancreatic tumors but affect less than 10/1,000,000 people per year [1], resulting in misdiagnosis as psychiatric or neurologic disease in up to 20% of patients, and they remain symptomatic for up to 2 years before diagnosis. A proper history and physical examination can help make the diagnosis based on the symptoms referred by the patient, and organic alterations should be considered part of the differential diagnosis in all patients with altered mental status. This case report intends to highlight the importance of integral care in medicine, making it key to approach the patient from multiple angles before reaching a definitive diagnosis.

## Case presentation

A 50-year-old Hispanic man with known history of obesity and hypertension presented with a 3-month history of abrupt episodes of weakness and altered mental status. He referred severe psychomotor symptoms as well as anxiety, aggressiveness, agitation, weakness, diaphoresis, and decreased visual acuity occurring at any time of the day and improved by eating. Upon further questioning, our patient, who worked as a truck driver and whose symptoms had reflected poorly in his workplace, also expressed overall discomfort with life and wishes of death due to the intensity of his symptoms. He had no history of tobacco smoking or excessive alcohol consumption. Medications taken prior to admission included candesartan 16 mg and hydrochlorothiazide (HCTZ) 12.5 mg once a day for hypertension.

During the following weeks, the symptoms increased in severity and frequency, with continued unremitting anxiety and weakness accompanied by loss of consciousness and extreme burning sensation in his entire body. After this acute episode, he was evaluated by a primary care physician in a local hospital and hospitalized for 4 days, where he continued to present recurrent episodes of irritability and aggression to self and others. He was discharged and referred for psychologic evaluation by mental health services at another institution. No specific diagnoses were given.

Upon release from mental evaluation, he was evaluated by a third physician, who performed a 3-hour postprandial blood glucose test which revealed a blood glucose level of 58 mg/dL. Suspecting an insulinoma, our patient was admitted to a third-level hospital, and upon arrival his vital signs were: afebrile (37 °C), heart rate of 93 beats per minute, blood pressure of 161/84 mmHg, respiratory rate of 19 breaths per minute, and oxygen saturation of 98% on room air. A physical examination revealed an oriented, well-nourished, and hydrated patient, without any stigmata on skin, a normocephalic and atraumatic head, and no masses or lesions. His eyes and ear, nose, and throat examinations were all within normal limits. His lungs were clear to auscultation without any added sounds, and heart sounds had a regular rate and rhythm without any murmurs, rubs, or gallops. An abdominal examination revealed a soft and nontender abdomen without any masses or organomegaly. Extremities showed no deformities, edema, skin discoloration, swelling, or tenderness. A neurological examination was within normal limits and showed an alert patient oriented to person, time, and place with fluent speech and comprehension. Cranial nerves II–XII were intact, with steady gait, with grossly intact sensation in all extremities. The reflexes were symmetric and 2+ at the biceps, triceps, knees, and ankles and had full strength in all extremities. Initial laboratory studies revealed a hemoglobin level of 15.7 g/dL (normal range, 13.7–17.5 g/dL), white blood cell count of 9.51 K/uL (normal range, 4.23–9.07), and calcium levels of 9.60 mg/dL (normal range, 8.42–10.22).

He was placed on the standard 72-hour fast to induce a hypoglycemic episode and became symptomatic after 20 hours. Further laboratory testing revealed blood glucose of 29 mg/dL (normal range, 100–125 mg/dL), increased insulin at 426 mIU/L (normal range, 2.6–24.9 mIU/L), and C-peptide levels of 12.83 ng/mL (normal range, 0.5–2.0 ng/mL). Sulfonylurea levels, abdominal ultrasound, and contrast magnetic resonance imaging (MRI) were also performed without findings. Prolactin, parathyroid hormone (PTH), albumin, and calcium levels, as well as MRI of his brain were normal, ruling out multiple endocrine neoplasia type 1 (MEN-1).

**Fig. 1:**
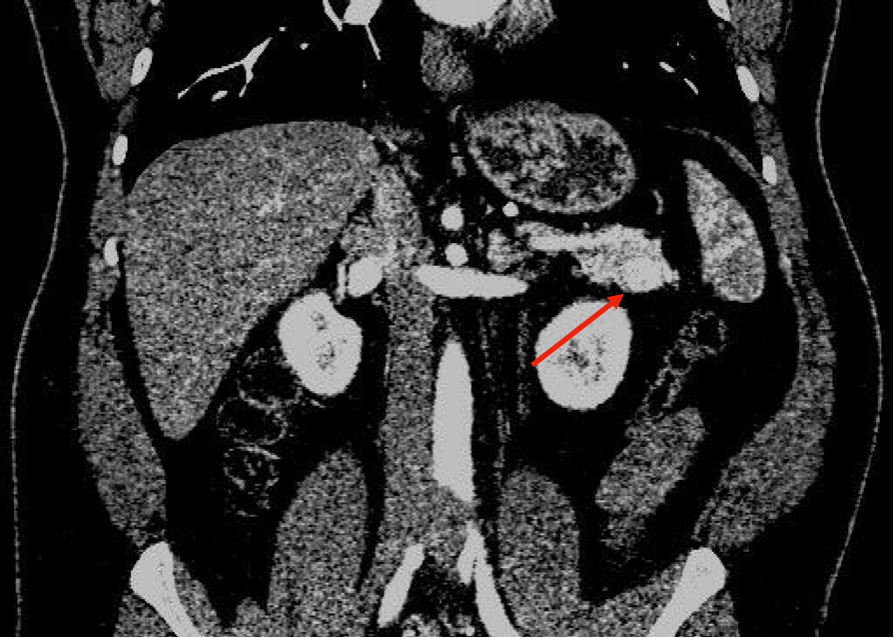
Triple phased abdominal computed tomography scan: arterial phase, coronal view. 1.8 × 1.6 hypervascular mass at the tail of pancreas (arrow)

**Fig. 2:**
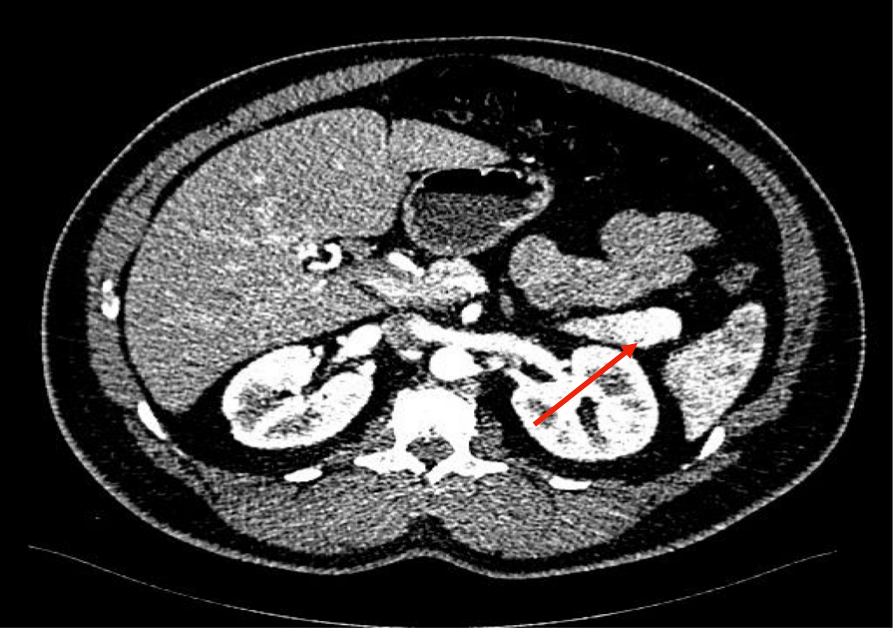
Triple phased abdominal computed tomography scan: arterial phase, axial view. 1.8 × 1.6 hypervascular mass at the tail of pancreas (arrow)

Prior to imaging, liver and renal function tests were performed, revealing alanine aminotransferase (ALT) levels of 27 U/L (normal range, 21–72), aspartate aminotransferase (AST) of 24 U/L (normal rang, 17–59), and creatinine levels of 0.89 mg/dL (normal range, 0.66–1.25). Urine analysis revealed no abnormal findings. After negative initial imaging, a triple phased computed tomography (CT) scan was performed, with an early arterial phase revealing a 1.8 × 1.6 cm hypervascular mass located at the tail of the pancreas (Figs. 1, 2)*.* Both kidneys had normal size and morphology, and slight hepatomegaly was shown as well as round nodules less than 10 mm in size in segments 3, 7, and 8.

At the completion of imaging studies, our patient was presented to the surgical department and scheduled for distal pancreatectomy in addition to a splenectomy due to the tumor’s close proximity to his spleen.

**Fig. 3:**
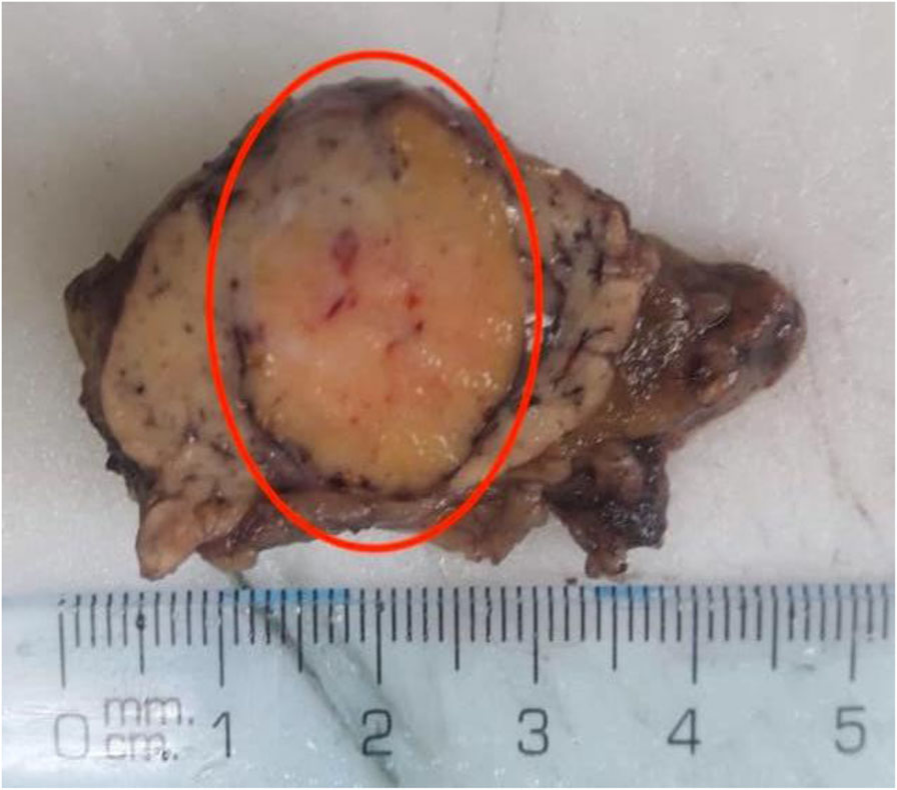
Macroscopic specimen from pancreatic tissue sample (red circle)

During the course of surgery an incidental appendectomy was also performed. A 4.5 × 3.5 × 1.5 cm specimen (Fig. 3) was collected during surgery and sent for pathologic analysis; the pathologic analysis reported the diagnosis of insulinoma, an encapsulated mass within the specimen that measured 1.5 × 1.5 × 1.0 cm and is consistent with the findings on CT scan.

**Fig. 4:**
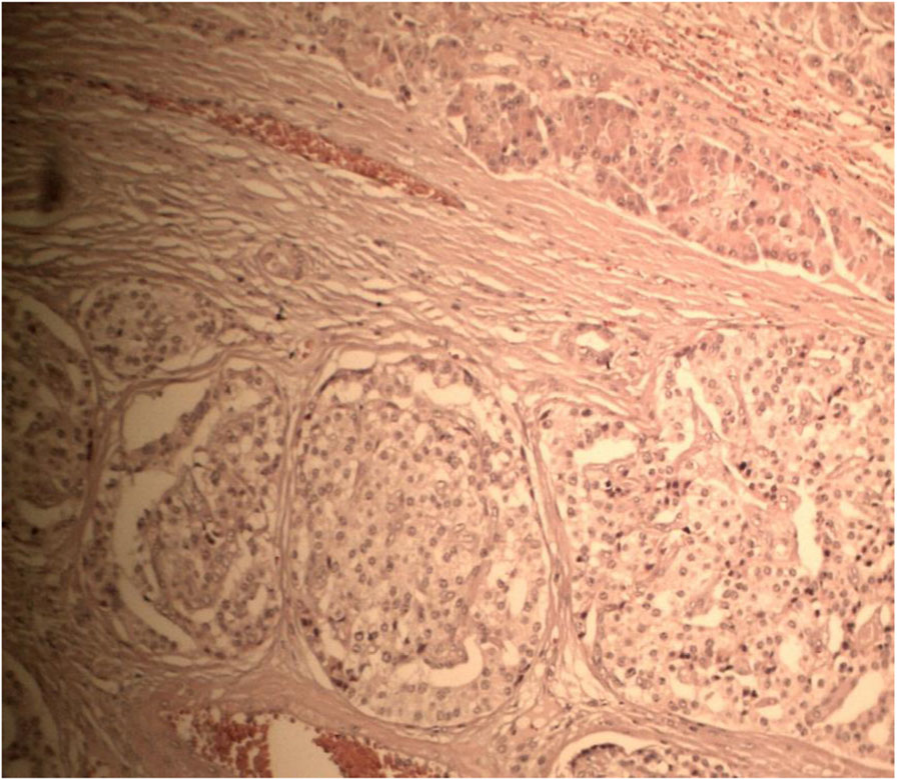
Microscopic specimen from pancreatic tissue with hematoxylin and eosin stain, × 300

Upon microscopic evaluation, histologic analysis revealed pancreatic tissue organized into islets within a vascularized stroma interspersed by hyaline tissue (Fig. 4). Surgical margins were clear of invasion and no metastasis was found in liver or abdominal lymph nodes.

### Discharge and follow-up

Our patient had no complications during the immediate postoperative period, in which he was tested for glucose (190 mg/dL, normal range < 126 mg/dL), lipase (207 U/L, normal range < 160 U/L), and calcium (8.5 mg/dL, normal range, 8.5–10.2 mg/dL), and administered omeprazole (40 mg intravenously once per day), insulin glargine (100 UI subcutaneous injection once per day), atenolol (100 mg orally once per day), HCTZ (25 mg orally once per day), metoclopramide (10 mg intravenously every 8 hours), nalbuphine (10 mg intravenously every 8 hours), and ceftriaxone (1 g intravenously every 12 hours). During the third postoperative day (POD), elevated respiratory rate, decreased oxygen saturation, and chest auscultation of diminished breaths sounds with rhonchi, pointed toward a diagnosis of left lobar pneumonia with pleural effusion. However, he was discharged on the fifth POD and prescribed ambulatory management and follow-up by endocrinology, pulmonology, and internal medicine units. On the ninth POD, he returned to our hospital with acute pain and serohematic secretions at the surgical site and was managed with drainage of the seroma and intravenously administered ketorolac (30 mg intravenously twice a day). A vaccination scheme was established on the 19th POD follow-up visit and started on the 18th POD with Prevnar-13® (pneumococcal 13-valent conjugate vaccine, diphtheria CRM_197_ protein), followed by Influenza (34th POD), and diphtheria and tetanus (DT) booster (53rd POD). On POD 21, he returned to our emergency room with acute pain, 10/10 intensity of both left quadrants. Laboratory results returned “within normal limits” and he was discharged on ambulatory management. He remains asymptomatic at 6-month follow-up and is currently being treated with ciprofibrate (100 mg orally once per day), amlodipine (10 mg orally once per day), enalapril (20 mg orally once per day), and aspirin (81 mg orally once per day)

## Discussion

Insulinomas are rare, yet they comprise the most common type of pancreatic tumor, and may present isolated or as part of the MEN-1 syndrome [1]. The incidence of this tumor is around 3–10 cases per million people every year, making it extremely uncommon in a population of 10 million people, the total national population of the setting described. To the best of our knowledge, the present case has been one of the few cases presented, diagnosed, and reported in the Dominican Republic [2]. Insulinomas are 90% benign, 90% solitary, 90% present in adult population, and 90% intrapancreatic [3]. The diagnosis of insulinoma is suggested by the presence of the Whipple triad in this patient: fasting hypoglycemia relieved by glucose ingestion and blood glucose levels below 45 mg/dL [3]. However, altered mentation has been reported in many instances as a common symptom of insulinoma, which may lead to confusion with psychiatric disease. These symptoms may take many different forms ranging from fatigue, confusion, poor concentration, altered behavior, irritability and “staring off”, to seizures and loss of consciousness" [4].

Other findings reported in cases of insulinoma are related to the adrenergic response to low glucose levels which may present as tremors, diaphoresis, blurry vision, and anxiety [5, 6]. Most of these symptoms present after a prolonged fast, and for in-patient diagnosis, a supervised 72-hour fast, which reports symptoms in 99% of patients within the completion of the fast [7]. Noninvasive imaging, such as MRI and CT as well as other invasive modalities (angiography) are used to localize the tumor once the clinical diagnosis is made [3].

The prompt exclusion of an organic disease as the cause of psychiatric symptoms is important in the reduction of further morbidity and mortality. Furthermore, it aids the patient to avoid exposure to prejudice or stigma that psychiatric disease may bring.

It is of upmost importance for primary care physicians to never neglect the basic principle that rules their practice, which is to assure that the patient receives a holistic approach toward all the aspects that concern care. A behavioral symptom could be the first clue for making the diagnosis of a systemic illness which can have a great variety of presentations including, but not limited to, behavioral changes.

At the time of evaluating a patient with psychiatric or behavior complaints it is important to inquire about symptoms related to a systemic cause of these symptoms and to take into account the resources that physicians can access during the time of evaluation.

The curative management of insulinoma is through the surgical removal of the tumor, either by pancreatic resection or enucleation and as the vast majority are benign, most patients are completely cured postoperatively [1]. Other nonsurgical techniques such as radiofrequency ablation, injection of octreotide, and endoscopic ultrasound-guided alcohol ablation have also been described for unresectable tumors and patients with complications who are unable to undergo surgery [3].

## Conclusion

We should keep in mind the importance of recording and reporting all signs and symptoms of diseases with low incidence, both locally and worldwide, in an attempt to provide the medical literature with a spectrum of red flags that will raise clinical suspicion and, therefore, be of aid to the physician at the time of diagnosis. Although not life threatening in the vast majority of cases, a tumor of such significance results in great distress to the patients and their families. This case reaffirms the importance of a holistic approach to diagnosis and treatment of our patients.
